# CRISPR in Sub-Saharan Africa: Applications and Education

**DOI:** 10.1016/j.tibtech.2018.07.012

**Published:** 2019-03

**Authors:** Christian E. Ogaugwu, Stanley O. Agbo, Modinat A. Adekoya

**Affiliations:** 1 Department of Microbiology and Molecular Genetics, University of California, Irvine, CA 92697-4500, USA; 2 Department of Animal and Environmental Biology, Federal University Oye-Ekiti, 371010 Ekiti State, Nigeria; 3 Department of Plant Science and Biotechnology, Federal University Oye-Ekiti, 371010 Ekiti State, Nigeria; 4 Laboratory website: http://sites.uci.edu/jameslab

**Keywords:** Africa, CRISPR gene editing, food security, disease control

## Abstract

Clustered regularly interspaced short palindromic repeats (CRISPR) technology has enabled genetic engineering feats previously considered impracticable, offering great hopes for solutions to problems facing society. We consider it timely to highlight how CRISPR can benefit public health, medicine, and agriculture in sub-Saharan Africa (SSA) and offer recommendations for successful implementation.

Human society has always faced two basic challenges: disease and hunger. Efforts to prevent/treat disease and achieve food security continue. Despite progress made, food insecurity and many diseases and pests persist; these problems have proved difficult to solve using available technologies. However, hopes are high for solutions to many of these challenges with the advent of CRISPR technology.

CRISPR technology is based on CRISPR-associated (Cas) proteins from bacterial immune systems. First developed using Cas9 endonuclease from *Streptococcus pyogenes* (spCas9), this system repurposed for gene editing utilizes a small guide-RNA (gRNA) with a changeable region of 20 nucleotide homology to a DNA target site to direct spCas9 to cleave the target DNA [1]. Other Cas proteins have subsequently been adapted for gene editing [2]. The technology is inexpensive and can be used for genetic modification in numerous organisms. Here, we explore potential applications of CRISPR in SSA and recommend steps necessary for successful implementation.

## How CRISPR Could Benefit Sub-Saharan Africa

Because of the prevalence of tropical diseases and pests, the public health, medical, and agricultural sectors in SSA would likely benefit greatly from CRISPR. Amongst tropical diseases, malaria ranks first for prevalence and is responsible for about half a million deaths yearlyi. Mosquitoes transmit malaria and other diseases, but control efforts have continued for decades with little success, owing to the complex biology of mosquitoes and limitations of existing strategies. Recently, CRISPR/Cas9-based gene drives (GDs) were developed for malaria vectors, *Anopheles stephensi* and *An. gambiae* 3, 4. With robust non-Mendelian inheritance, these strains could spread antimalarial genes or suppress generations of wild populations. Although proofs-of-concept yet to undergo larger-scale tests in containment or in the field, their laboratory successes indicate the strong potential of CRISPR-based GDs for the elimination of malaria [5]. However, African malaria is complex and involves *An. gambiae, An. colluzzi, An. funestus*, and other species. A holistic malaria control approach would require GDs for these other species too, perhaps in combination with compatible existing approaches. GDs also might control other disease vectors like *Aedes aegypti*, which transmits dengue, chikungunya, and Zika viruses, for which good vaccines or cures are currently lacking. Likewise, population-suppressing CRISPR-based GDs would be suitable for mitigating destructive invasive pests in the region that urgently need but lack efficacious, cost-effective, and sustainable area-wide control, especially the fall armyworm *Spodoptera frugiperda*, which wreaks havoc on major staplesii, and *Bactrocera dorsalis* (synonym *invadens*), which destroys fruitsiii.

Besides GDs and other potential applications (Box 1), CRISPR is particularly suitable for curing prevalent genetic diseases in Africa, such as sickle-cell anemia (SCA) (Box 2), which had no cure until recently when Ribeil and colleagues demonstrated resolution of symptoms in a patient via lentiviral gene therapy [6]. Although a breakthrough, this method is expensive, lengthy, and solely postnatal, amidst concerns about post-treatment infections. Proofs-of-principle in mice and preimplantation human embryos revealed the possibilities to correct β-thalassemia (related to SCA) and other pathogenic mutations using CRISPR-Cas9 alone or combined with other methods 7, 8, supporting the view that CRISPR could become a method of choice for efficient and cost-effective pre- or postnatal gene therapy for SCA in SSA.

Box 1 **Other Potential Uses of CRISPR in SSA**

CRISPR could facilitate vaccine production against tropical diseases like malaria, presently constrained by inadequate donor supportv and complexity of parasites. Irradiated sporozoites confer immunity against malaria, but vaccines using them face mass production challenges as the amount ejected by mosquitoes is low. Bites from mosquitoes harboring irradiated sporozoites are another option, albeit unpleasant and objectionable. These circumstances necessitate adoption of other strategies for malaria vaccines. Mosquito innate immune systems fight *Plasmodium* infections with several genes playing roles, and their silencing via RNAi in anophelines modulate immune pathways for or against infection [13]. However, RNA is transient, and RNAi sometimes fails to fully suppress gene expression. CRISPR offers permanent heritable gene silencing and is ideally better suited than RNAi to engineer mosquitoes with copious sporozoite production for vaccines. Crops and animals in SSA also face many pests and pathogens. Breeding programs attempt to tackle these but have so far achieved little. CRISPR could help breeders in SSA to produce improved farm animals or crops with pest/disease resistance or other desirable traits. Exploitation of CRISPR to modify different plants has been demonstrated and the recent genome editing in the tropical staple, cassava [14], sets the stage for greater attempts at realizing food security in SSA.

Alt-text: Box 1

Box 2 **Sickle-Cell Anemia**

SCA is a genetically inherited disease that is widespread throughout SSA, which has about 80% of known cases worldwidevi. Characterized by anemia, intense pain, chest syndromes, and hemolytic crisis, resulting from abnormal sickle-shaped red blood cells, the disease is caused by recessive mutant alleles of the oxygen carrier, hemoglobin S (HbS) inherited from parents by offspring. The normal allele, hemoglobin A (HbA), is dominant. Inheritance of a HbA allele together with a HbS allele leads to a carrier individual who does not suffer from the disease. The heterozygous condition confers some protection against *Plasmodium* parasites that cause malaria; a factor that may have acted as a selection pressure for the HbS allele. Offspring who inherit HbS alleles from both parents become sufferers and have symptoms of the disease. Genetically, HbS has a single nucleotide mutation (A to T) in the nucleotide sequence of β-globin gene, which converts glutamic acid at position 7 in the normal β-globin allele to valine. It is this defect that makes red blood cells of HbS aggregate and adopt sickle shapes instead of round shapes under low oxygen conditions [15]. Correction of this mutation would abolish the disease in sufferers.

Alt-text: Box 2

## Key Factors and Recommendations for Success of CRISPR in Sub-Saharan Africa

Factors crucial for successful CRISPR implementation in SSA deserve consideration. With the imminent application of GDs against malaria mosquitoes in this region, recommendations are offered here to guide such campaigns for future CRISPR applications.

### Efficacy and Benefits with Less Harm

Success for CRISPR in SSA will depend on two major factors: demonstrating efficacy to achieve desired outcomes and providing evidence that benefits outweigh any potential risks. These should be adequately addressed for any potential CRISPR application, especially those designed for field use.

For GD mosquitoes, field testing on islands off the continental coast of SSA will be a good first step to establish how it might work in the field and investigate any potential risks, before considering the complex continental mainland. Climatic, ecological, and other conditions on the islands might resemble those in the mainland where malaria control programs have their main focus. Being geographically isolated, they fit well for contained/open field trial programs. In addition, islands tend to have low population diversities, whereas the continental mainland has recently been shown to have a high genetic diversity of the African malaria mosquito, *An. gambiae* [9]. These factors suggest that islands would be the best choice for the first field test. Nevertheless, contained cage trials could be performed in appropriate research facilities elsewhere on the mainland if unforeseen circumstances complicate finding a good island for a field test. The high genetic diversity among *An. gambiae* [9] also calls for cautious genetic analysis of populations/subpopulations prior to GD development/field tests to ensure that their efficacy and success are not jeopardized by polymorphisms. Postanalysis, genomic target sequences for gRNAs can be designed to match those in the chosen population/subpopulation. Genomic sites with high variability within the same population/subpopulation should be avoided, while highly conserved sites can be selected. Targeting multiple conserved genomic sites could also minimize chances that organisms might develop resistance to GDs. Furthermore, present GD mosquitoes were generated using progenitors colonized in laboratories for several years and may not be well suited to the environment of SSA. To circumvent this, GD mosquitoes should be developed using recently colonized mosquitoes caught from prospective release locations. Although best for the future, developing GDs in sub-Saharan African laboratories [5] using recently colonized mosquitoes from prospective locations will likely have to deal with the scientific capabilities within the region. Alternatively, GD mosquitoes developed in laboratories elsewhere could be used [5] and outcrossed to wild-caught ones from locations in SSA. This latter option should be easier but would confront international restrictions.

In treating human diseases, a centralized hospital-centric approach rather than a decentralized clinic-based approach may be best to ensure adherence to regulations and better assessment of treatment outcomes. CRISPR usage against SCA will likely fare better in SSA than other potential applications, given that consent and implementation is individual dependent. Government or public approval may vary unpredictably, but if developed with proven efficacy this method could gain popularity among sufferers. The distress caused by the disease is enormous and most sufferers would try novel approaches to end their ordeal if the expected benefits outweigh the perceived risks.

Every technology has some risks, and CRISPR is no exception. Off-target effects occur in modified organisms and need careful evaluation. Bioinformatics tools can be used to predict or minimize/avoid them. However, horizontal gene transfer and potential crossover harm to humans and the environment are major concerns. The odds do not favor cross-activity of CRISPR to engender unintended harm: gRNAs matching DNA target sites adjacent to recognizable protospacer adjacent motifs are required besides the presence of functional nucleases, RNAs degrade easily and degraded gRNAs cannot be utilized, and targeted sequences might not match in different species/organisms. Nonetheless, CRISPR is still a new technology not yet thoroughly understood, so objective scientific studies assessing risks/harms and establishing acceptable thresholds are needed. For such studies, expert workshop reports [10] would be invaluable guides. Knowledge of persistence and the activity/functionality window of CRISPR in organisms and the environment would also benefit risk assessment. Incorporation of recall approaches [11] or use of controllable CRISPR nucleases in GDs and other applications would ease their removal/stoppage if necessary.

### Education with Transparency

Transparency and education of local people are other factors that will affect successful implementation of CRISPR in SSA. Due to societal vulnerability to rumors and conspiracy theories if knowledge of new programs or technologies is lackingiv, it is important to create adequate awareness and to educate local communities. Education should enable informed consent or refusal and could also propel local people to work towards the success of a CRISPR program in their communities rather than against it. Often, unpredictable national or local politics arise in different sectors and could forestall CRISPR implementation. Thus, consultations with relevant stakeholders/policymakers following proper channels will be paramount. This should advance implementation since stakeholders would ideally have knowledge of other ongoing programs in their constituencies and whether such programs could aid or interfere with an intended CRISPR approach. For example, the release of GD mosquitoes to spread malaria-resistant genes [3] may not be compatible with ongoing insecticide spray programs, as the sprays would kill released GD and most wild mosquitoes, while insecticides might aid population suppression with GD mosquitoes designed for eradicating populations [4]. To ease outreach, organization, and implementation, national or local committees comprising experts, stakeholders, and community members should be constituted. These would further awareness/education since community members in committees would serve to communicate with their communities in local languages. Further community engagement activities can be adapted from successful programs like the transgenic mosquito field trial in Brazil [12]. Involvement of local scientists in development of CRISPR-based strategies for their societies would positively impact the transparency of the program, give communities a sense of belonging, and boost their trust in the programs.

## Concluding Remarks

CRISPR has tremendous potential and can be deployed against a wide array of problems. Its implementation in SSA will doubtless encounter challenges, some of which we have highlighted and recommended solutions. Other hurdles for CRISPR include international/national regulations and ethical guidelines. Care should be taken to ensure that the first CRISPR application in SSA succeeds. Being foremost amongst other potential CRISPR applications, success for GD mosquitoes in curbing malaria, even on small islands, would likely be the prelude to the eradication of malaria on the continent. Moreover, this would open the path for implementation of other potential CRISPR applications and lead to much reduced prevalence of disease and pests in the region.

## Resources

^i^www.who.int/malaria/publications/world-malaria-report-2017/report/en/

^ii^www.fao.org/food-chain-crisis/how-we-work/plant-protection/fallarmyworm/en/

^iii^https://en.wikipedia.org/wiki/Bactrocera_invadens

^iv^www.nbcnews.com/id/15005238/ns/health-infectious_diseases/t/rumors-cause-resistance-vaccines-nigeria/#.Wzx9vcInbIU

^v^www.malariavaccine.org/malaria-and-vaccines/need-vaccine

^vi^https://en.wikipedia.org/wiki/Sickle_cell_disease
